# Validation of the Muscle Dysmorphic Disorder Inventory (MDDI) among Italian Women Practicing Bodybuilding and Powerlifting and in Women Practicing Physical Exercise

**DOI:** 10.3390/ijerph19159487

**Published:** 2022-08-02

**Authors:** Silvia Cerea, Matteo Giraldo, Corrado Caudek, Gioia Bottesi, Antonio Paoli, Marta Ghisi

**Affiliations:** 1 Department of General Psychology, University of Padova, 35131 Padova, Italy; matteo.giraldo@unipd.it (M.G.); gioia.bottesi@unipd.it (G.B.); marta.ghisi@unipd.it (M.G.); 2 Department of Biomedical Sciences, University of Padova, 35131 Padova, Italy; antonio.paoli@unipd.it; 3 Neurosciences, Psychology, Drug Research and Child Health (NEUROFARBA), University of Florence, 50139 Florence, Italy; corrado.caudek@unifi.it; 4 Hospital Psychology Unit, University-Hospital of Padova, 35128 Padova, Italy

**Keywords:** muscle dysmorphia, muscle dysmorphic disorder inventory, women, physical exercise, bodybuilding, powerlifting

## Abstract

Studies pertaining to muscle dysmorphia (MD) have concentrated the most on males. However, a new body ideal for women is emerging: a very toned, athletic body with flat, smooth muscles. The emphasis on the level of muscularity represents a contribution to the growth of MD symptoms in women. The aim of this study was to evaluate the factorial structure and psychometric properties of the muscle dysmorphic disorder inventory (MDDI) in two samples of physically active Italian women. One-hundred and sixty-five women practicing non-competing bodybuilding/powerlifting and 353 women practicing physical exercise completed the MDDI and measures of features associated with MD. Findings of the confirmatory factor analysis showed a three-factor structure with acceptable fit and invariant across groups. Omega coefficients revealed adequate internal consistency for all the scales and for the total score of the MDDI. Furthermore, convergent and divergent validity as well as retest reliability emerged to be good. MDDI represents a reliable measure of MD symptoms in physically active Italian women.

## 1. Introduction

Muscle dysmorphia (MD) is a psychological disorder characterized by the fear of being too thin and by perceiving oneself as small and weak, even when one is actually large and muscular [1]. MD is defined by three main criteria: preoccupation with the idea that one’s body is not sufficiently lean and muscular; negative beliefs about one’s body that lead to body avoidance or anxiety; and the interference of these two aspects in social and/or occupational functioning [2]. MD is also characterized by a drive for muscularity, which leads to behaviors such as disordered eating practices; intense anxiety or avoidance of situations where one’s body is exposed; excessive training despite adverse physical consequences; forgoing social, occupational, or recreational activities to maintain one’s workout and diet schedule; significant distress and/or social or occupational impairment; and anabolic androgenic steroids (AAS) and hormones use [3,4,5]. Furthermore, MD symptoms are associated with depressive and anxiety symptoms, social physique anxiety, and low self-esteem [6,7,8].

Studies pertaining to MD have concentrated the most on men. Consistently, a systematic review has shown that, in 34 studies pertaining to MD, only 6 have included women in their sample [9]. However, a new body ideal for women is emerging and taking the place of the thin ideal: a very toned, athletic body with flat, smooth muscles [10,11]. The new emphasis on females’ muscularity represents a contribution to the growth of MD symptoms in women, highlighting the importance of further investigating this phenomenon in females [9] by means of valid and reliable self-report measures. The investigation by Readdy, Watkins, and Cardinal [12] revealed similar levels of MD symptoms between male and female college students. Furthermore, Gruber and Pope [13] showed that, in a sample of 75 female bodybuilders, 66 experienced symptoms of MD and Leone [14] described, in a case study, a 23-year-old woman with MD symptomatology. Despite these studies investigating MD symptoms in females, the majority enrolled a small number of participants, focused on college students, and were conducted in the United States. Furthermore, most of these studies did not employ validated self-report measures to investigate MD symptoms in women, which represent an essential prerequisite for research in the MD field, as well as for screening in the clinical context [15]. 

Of several questionnaires that have been developed to measure MD symptoms, one of the most commonly used is the muscle dysmorphic disorder inventory (MDDI), which is the only one currently available assessing functional impairment, a core component of the diagnosis of MD [2]. The MDDI [16] is a 13-item self-report measure including three scales assessing clinical features associated with MD: drive for size (DFS), appearance intolerance (AI), and functional impairment (FI). The questionnaire has been validated in a sample of 237 male weightlifters and showed a three-factor solution after a Principal component analysis (PCA) [16]. The total variance explained was 63.02%; the three scale variances explained were: DFS (28.50%), AI (18.34%), and FI (16.19%). The original MDDI showed a good internal consistency (DFS: α = 0.85; AI: α = 0.77; FI: α = 0.80; total score: α = 0.81) and good 1-week test–retest reliability (*r* = 0.87). Divergent and convergent validity were also good. Based on these results, MDDI emerged as a short and reliable measure of MD symptoms, and it is currently considered the gold standard measure for MD symptomatology [17]. In accordance, the MDDI has been validated in several languages. However, most of the validation studies have been conducted with male samples or with mixed-gender samples, except for a very recent study conducted with a sample of Brazilian women [18], which replicated the original three-factor structure of the questionnaire. However, this study did not examine the test–retest reliability of the questionnaire.

Pertaining to male samples, Santarnecchi and Déttore [19] validated the Italian version of the MDDI in a sample of 120 male bodybuilders (60 competing and 60 non-competing) and 60 non-training males. The Italian version of the MDDI revealed, through a PCA, the same factorial structure of the original version [16] in competing bodybuilders. The total variance explained was 66.22%; the three factors, DFS, FI, and AI, explained, respectively, 39.32%, 15.37%, and 11.52% of the total variance. For non-competing bodybuilders and non-training males, the PCA revealed two different four-dimension solutions. In non-competing bodybuilders (74.59% of total explained variance), the fourth factor emerged to be the result of the AI scale split into two different factors. In non-training individuals (85.15% of the total explained variance), the fourth factor was a balanced spin-off of the original tripartite factorial composition. Both convergent and divergent validity were good, as well as internal consistency, with the exception of the AI scale (DFS: α = 0.80; FI: α = 0.81; AI: α = 0.45; total score: α = 0.85). Authors also reported a good 3-week test–retest reliability (DFS: *r* = 0.83–0.97; AI: *r* = 0.79–0.83; FI: *r* = 0.82–0.85; total score: *r* = 0.62–0.99) [19]. Similar results emerged in the study by Sepúlveda and colleagues [20] among 298 Spanish male university students and in the study by Compte and colleagues [17] in two samples of Argentinian men who exercise (276 weightlifters and 275 CrossFit users). 

Pertaining to mixed-gender samples, the MDDI has been validated by Galiana, Badenes-Ribera, and Fuentes [21] in a sample of 279 Spanish psychology university students (72.4% women). The Spanish version of the MDDI showed a factorial structure similar to the one which emerged in the original validation study by means of an exploratory factor analysis (EFA); however, the DFS scale accounted for the least percentage of variance explained among factors (19.38%), contrary to what has been observed in the original MDDI. Furthermore, the MDDI has been validated in Germany in a mixed-gender sample [22]. The German version of the MDDI replicated the factorial structure of the original version of the questionnaire and was found to be invariant across genders [22]. 

As previously described, there has been a lack of studies on MDDI validation and on its psychometric properties among females, despite the presence of women suffering from MD having been reported to be growing, especially in the bodybuilding context [23,24,25] and among women who lift weight [26]. Ensuring that MDDI is reliable in a specific population, such as physically active women, represents an essential prerequisite for research in the MD field [15], especially given the increased risk of MD symptoms in women due to changes in the female body ideal that place greater emphasis on being toned, fit, or athletic, fostering the development of MD symptoms [10,11,27]. The only study that performed a psychometric evaluation of the MDDI in females [18] did not examine the test–retest reliability of the questionnaire; furthermore, the authors did not investigate the practice of physical activity. However, physically active women may be particularly at risk of experiencing MD symptoms, since physical exercise might be employed as a vehicle to improve/maintain body image [28]. Therefore, the validation of MDDI in women involved in physical activity is crucial to deeper investigate MD symptoms in this population. In this study, first data on the factorial structure and psychometric properties of the Santarnecchi and Dèttore [19] version of the MDDI were provided in two Italian female samples: (1) women practicing non-competing bodybuilding/powerlifting and (2) women practicing physical exercise other than bodybuilding/powerlifting. 

In the first place, the current study aimed to explore the factorial structure of the MDDI by performing two confirmatory factor analyses (CFAs) that tested two different models: a unidimensional model and a three-factor model. In accordance with previous studies that have explored the MDDI factorial structure in mixed-gender samples [21,22], a three-factor structure with a higher-order factor is expected. Measurement invariance across women practicing non-competing bodybuilding/powerlifting and women practicing physical exercise other than bodybuilding/powerlifting was also evaluated. 

Furthermore, the internal consistency, temporal stability, and convergent/divergent validity of the MDDI were explored in both samples. Pertaining to convergent/divergent validity, measures of self-esteem, social phobia symptoms, and general distress were included in the current study. These constructs were selected based on their significant associations with MD [6,7,8]. Evidence of convergent/divergent validity would be demonstrated through: (1) positive associations between MDDI scores and social phobia symptoms, and general distress; and (2) negative associations with self-esteem. In general, good psychometrics properties (i.e., good internal consistency, temporal stability, and convergent/divergent validity) for the questionnaire are expected, in accordance with previous studies [16,17,19,20,21,22].

Lastly, novel issues scarcely investigated in previous studies were addressed to expand knowledge on MD symptoms in physically active women. First, the associations between the three original MDDI scales and socio-demographic features (age and education), BMI, and training features (starting date of training and weekly training) were analyzed, speculating that small correlations between socio-demographic features and MDDI scores would emerge. Pertaining to BMI, negative associations with the DFS scale were expected, in accordance with previous studies suggesting that a low BMI is associated with the desire to get bigger and more muscular [29]. Pertaining to training features, positive associations between the FI subscale and the weekly training are expected, as items are focused on the distress experienced during off-training days and social impairment due to training schedule. 

## 2. Materials and Methods

### 2.1. Participants and Procedure

Two samples were recruited for the study: a main sample and a retest sample. Inclusion criteria of the study were at least 18 years of age, practicing physical exercise (training at least once a week), and being female. The only exclusion criterion of the study was training less than once a week. Participants were recruited online via advertisements placed on social media sites (i.e., Instagram and Facebook) and on bodybuilding, powerlifting, and fitness forums. Participants were invited to participate in a study about “body image and physical activity”. Participants identified themselves as non-competing bodybuilders/powerlifters or as women practicing physical exercise other than bodybuilding/powerlifting. Individuals participated on a voluntary basis and provided their informed consent by clicking agreement before starting to complete the survey about body image and physical activity; participants were also informed about the possibility to withdraw from the survey at any stage without explanation. After providing informed consent, participants completed a schedule collecting socio-demographic information, training information, and self-report measures; responses were saved on the Google Drive server. Participants took approximately 25 min to complete the survey. To ensure that no participant completed the survey more than once, personal codes provided by participants (consisting of the first letters of their first and last names followed by their year of birth) were examined, as well as internet protocol (IP) addresses.

Participants did not receive any kind of reimbursement for their participation in the study. The study was conducted in accordance with the Declaration of Helsinki and approved by the Ethics Committee for the Psychological Research of the University of Padova.

### 2.2. Main Sample

Participants of the main sample numbered five hundred and eighteen women. A total of 165 identified themselves as non-competing bodybuilders or as non-competing powerlifters, whereas 353 identified themselves as women practicing physical exercise other than bodybuilding/powerlifting. Participants ranged in age from 18 to 58 years, in education from 5 to 21 years (accordingly with the Italian school system, 5 years of education correspond to primary school, 8 to middle school, 13 to high school, 16 to bachelor’s degree, 18 to master’s degree, and 21 to PhD), and in self-reported body mass index (BMI) from 19.24 to 24.06 kg/m^2^. Participants started to exercise 1 month to 15 years ago and referred to exercise 1 to 7 times a week. Overall sample (*n* = 518) descriptive and group differences (non-competing bodybuilding/powerlifting group *vs.* physical exercise group) in socio-demographic and training features are reported in Table 1. 

### 2.3. Retest Sample

A second sample of participants made up of 100 women was recruited and asked to complete the MDDI at T0 and after 1 month (T1) to test the temporal stability of the MDDI. Inclusion criteria were the same as for the main sample. Fifty women identified themselves as non-competing bodybuilders or as non-competing powerlifters, whereas the other 50 identified themselves as women practicing physical exercise other than bodybuilding/powerlifting. Participants ranged in age from 18 to 48 years, in education from 8 to 22 years, and in self-reported BMI from 16.53 to 35.88 kg/m^2^. Participants started to exercise 1 month to 21 years ago and referred to exercise 1 to 6 times a week. Retest sample (*n* = 100) descriptive in socio-demographic and training features are reported in Table 2.

### 2.4. Measures

The *socio-demographic and training information schedule*: employed to assess socio-demographic and training information of participants such as gender, age, years of education, marital status, employment, self-reported weight and height, months of training, and weekly frequency of training. Height and weight data were used to compute self-reported BMI as kg/m^2^. 

The *muscle dysmorphic disorder inventory* (MDDI; [16,19]): 13-item measure that assesses symptoms of muscle dysmorphia on a five-point Likert scale (from 1 = never to 5 = always). The MDDI includes a total score and three subscales: drive for size (DFS), appearance intolerance (AI), and functional impairment (FI). The DFS scale includes questions about thoughts of being smaller, weaker, and less muscular than desired; the AI scale is composed of questions about negative thoughts and anxiety related to the body; and the FI scale consists of questions about excessive exercise and behaviors to maintain the exercise routine and avoidance of social situations.

The *Rosenberg self-esteem scale* (RSES; [30,31]): self-report questionnaire made up of 10 items rated on a four-point Likert scale assessing self-esteem. Good internal consistency has been reported for the Italian version of the RSES (α = 0.84) [31]. In our sample, internal consistency was *ω* = 0.92 (95% CI = 0.90–0.94) for the non-competing bodybuilders/power-lifters subsample and *ω* = 0.92 (95% CI = 0.91–0.93) for the physical exercise subsample.

The *social phobia scale* (SPS; [32,33]): 20-item measure assessing situations that involve being observed by others. Items are rated on a five-point Likert scale. The Italian version of the SPS showed good psychometric properties (internal consistency: α = 0.87; 30-day test–retest reliability: *r* = 0.87; [33]). In our sample, internal consistency was *ω* = 0.94 (95% CI = 0.93–0.95) for the non-competing bodybuilders/power-lifters subsample and *ω* = 0.94 (95% CI = 0.93–0.95) for the physical exercise subsample.

The *depression anxiety stress scale-21* (DASS-21; [34,35]): 21-item self-report questionnaire assessing depression, anxiety, and stress on a four-point Likert scale. The Italian version proved to be reliable, with internal consistency values ranging from α = 0.74 to α = 0.90 in a community sample [35]. Given that findings of the Italian version suggested that the use of a total score could be more appropriate than calculating the three scale scores separately [35], only the total score of the questionnaire was considered for the study. In our sample, internal consistency was *ω* = 0.95 (95% CI = 0.94–0.96) for the non-competing bodybuilders/power-lifters subsample and *ω* = 0.95 (95% CI = 0.95–0.96) for the physical exercise subsample.

### 2.5. Analytic Strategy

There were no missing data in our dataset. To examine the factorial structure of the MDDI, two CFAs were performed using the R software package (version 3.4.4; [36]), specifically the R package lavaan [37]; the weighted least squares mean and variance (WLSMV) estimator was employed to allow the modeling of ordinal data in CFAs. To determine the fit of the CFA models, the *χ*^2^ test statistic, the comparative fit index (CFI), the Tucker–Lewis index (TLI), the root mean square error of approximation (RMSEA), and the standardized root mean square residual (SRMR) were considered. Generally, CFI and TLI values larger than 0.90 are taken to indicate acceptable fit, although values greater than 0.95 are desirable [38]. RMSEA values lower than 0.05 indicate close fit, values between 0.05 and 0.08 indicate acceptable fit, values between 0.08 and 0.10 indicate mediocre fit, and values greater than 0.10 indicate poor fit [39]. SRMR values range from 0 to 1.0, with well-fitting models obtaining values smaller than 0.05 [40]; however, values as high as 0.08 are deemed acceptable [41]. Next, the models were compared using both a qualitative evaluation of the fit indices and the ΔCFI criterion. As recommended by [42], if the difference in the CFIs between two nested models (ΔCFI) is smaller than |0.01|, the hypothesis of no differences in fit between the two competing models should not be rejected. 

Internal consistency in both subsamples was assessed using McDonald’s omega and its associated 95% CI [43], with values greater than 0.70 reflecting adequate internal reliability [44].

Correlations among the MDDI scales and convergent/divergent validity were assessed with Pearson’s correlation analyses (*r*), separately for non-competing bodybuilders/powerlifters and women practicing physical exercise. Furthermore, Pearson’s correlation analysis (*r*) was calculated to investigate the association between MDDI scores and socio-demographic, BMI, and training features separately for non-competing bodybuilders/powerlifters and women practicing physical exercise. Lastly, Pearson’s correlation analysis (*r*) was conducted to assess MDDI test–retest reliability. Based on Cohen [45], values ≤ 10 were considered weak, ~0.30 were considered moderate, and ~0.50 were considered strong correlations.

Group differences in socio-demographic information, training data, and in MDDI scores were investigated with independent-samples *t*-tests. Bonferroni correction was employed to adjust probability (*p*) values because of the increased risk of a type I error when making multiple comparisons; in accordance, *ps* of 0.005 were considered significant.

Statistical analyses were conducted in IBM SPSS Statistics (version 25), except for CFAs and *ω* and associated 95% CI that were computed with the R software package (version 3.4.4; [36]); specifically, the R package lavaan [37] was employed for CFAs, while the R package “userfriendlyscience” [46] was used for *ω* and associated 95% CI.

## 3. Results

### 3.1. Confirmatory Factor Analysis

**Figure 1:**
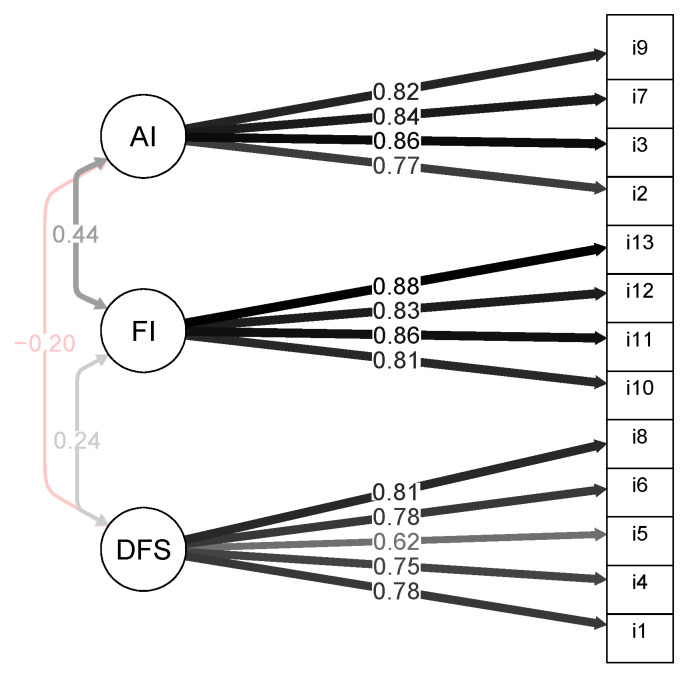
Path diagram for the three-dimensional model of the muscle dysmorphic disorder inventory (MDDI) in physically active Italian women.

Parallel analysis indicated that three factors should be extracted, which accounted for the 63.84% of the total variance. To test the usefulness of separate measures for drive for size (DFS), functional impairment (FI), and appearance intolerance (AI), CFAs were employed to compare a three-factor and a one-factor model for the MDDI. The three-factor model produced adequate fit measures (*χ*^2^(62) = 276.988, CFI = 0.982, TLI = 0.977, RMSEA = 0.082, SRMR = 0.085) (see Figure 1), whereas the one-factor model did not (*χ*^2^(3) = 785.55, *p* = 0.0001).

### 3.2. Group Invariance

Factorial invariance was tested by comparing a series of increasingly restrictive models with progressively more restrictive hypotheses about equality across groups [47]. Configural (equivalence of model form), invariance of thresholds (measurement invariance for ordinal measures), metric (equivalence of factor loadings), and scalar (equivalence of item intercepts) invariance models produced acceptable fit indices (CFI = 0.953–0.956, RMSEA = 0.078–0.085). In terms of the ΔCFI < 0.01 criterion (e.g., [48]), no deterioration of fit with the constraints of configural, invariance of thresholds, metric, and scalar invariance models were found (Table 3). Therefore, the MDDI obtained scale invariance across the non-competing bodybuilding/powerlifting and the physical exercise groups.

### 3.3. Internal Consistency

Internal consistency coefficients were adequate in both the non-competing bodybuilding/powerlifting subsample (MDDI total score: *ω* = 0.79, 95% CI = 0.74–0.84; DFS: *ω* = 0.82, 95% CI = 0.78–0.87; AI: *ω* = 0.87, 95% CI = 0.83–0.90; FI: *ω* = 0.88, 95% CI = 0.85–0.91) and in the physical exercise subsample (MDDI total score: *ω* = 0.76, 95% CI = 0.72–0.80; DFS: *ω* = 0.71, 95% CI = 0.66–0.76; AI: *ω* = 0.86, 95% CI = 0.84–0.88; FI: *ω* = 0.82, 95% CI = 0.79–0.85). 

### 3.4. Correlations among the MDDI Scales 

In both subsamples, the total score of the MDDI showed positive correlations with all the MDDI scales (non-competing bodybuilding/powerlifting subsample: *rs* ranging from 0.57 to 0.81; physical exercise subsample: *rs* ranging from 0.43 to 0.80). Correlations between scales were significant in both subsamples with the exception of the correlations between the DFS scale and the AI scale in both groups (non-competing bodybuilding/powerlifting subsample: *r* = −0.15; *p* = 0.06; physical exercise subsample: *r* = −0.09; *p* = 0.07). Correlations are reported in Table 4. 

### 3.5. Convergent and Divergent Validity 

The MDDI total score was significantly correlated with the other self-report measures; overall, correlations were moderate in magnitude. To note, the DFS scale did not correlate with any of the other employed self-report measures in the non-competing bodybuilding/powerlifting subsample. With respect to the physical exercise subsample, no correlations emerged among the DFS subscale and the RSES. Results are reported in Table 4. 

### 3.6. Associations between MDDI Scores and Socio-Demographic (Age, Education), BMI, and Training Features (Starting Date of Training and Weekly Training)

Overall, most of the observed correlations were small in magnitude. Significant correlations between the MDDI total score and age emerged in both groups, whereas a significant correlation with weekly training emerged only in the non-competing bodybuilding/powerlifting subsample. The FI scale correlated with weekly training in both groups (moderately in the non-competing bodybuilding/powerlifting group; poorly in women practicing physical exercise), whereas it correlated with the starting date of training only in the non-competing bodybuilding/powerlifting subsample and with age only in women practicing physical exercise. The AI scale showed significant medium-range correlations with BMI in both groups, whereas a significant but small correlation with the starting date of training emerged only in women practicing physical exercise. Lastly, the DFS scale emerged to be moderately correlated with BMI in both groups, whereas a significant small correlation with age emerged only in women practicing physical exercise. The results are reported in Table 4.

### 3.7. Between-Group Comparisons

Significant differences between groups emerged in all the MDDI scales, except for the AI scale. In all cases, non-competing bodybuilders/powerlifters scored higher than women practicing physical exercise. Results are presented in Table 5. 

### 3.8. Retest Reliability

One-month retest reliability in 100 women (non-competing bodybuilding/powerlifting subsample: *n* = 50; physically exercise subsample: *n* = 50) was excellent. Non-competing bodybuilding/powerlifting subsample: MDDI FI scale: *r* = 0.88; *p* < 0.001; MDDI AI scale: *r* = 0.94; *p* < 0.001; MDDI DFS scale: *r* = 0.90; *p* < 0.001; MDDI total score: *r* = 0.93; *p* < 0.001. Physical exercise subsample: MDDI FI scale: *r* = 0.93; *p* < 0.001; MDDI AI scale: *r* = 0.92; *p* < 0.001; MDDI DFS scale: *r* = 0.92; *p* < 0.001; MDDI total score: *r* = 0.95; *p* < 0.001.

## 4. Discussion

The aim of the current study was to evaluate the factorial structure and psychometric properties of the MDDI in two subsamples of Italian women: women practicing non-competing bodybuilding/powerlifting and women practicing physical exercise. Our results provide evidence that the MDDI is a valid and reliable measure for use in physically active Italian women.

First, the factorial structure of the MDDI was evaluated by performing two CFAs that tested two different models: (1) a unidimensional model and (2) a three-factor model. In line with our hypothesis, the results indicated that the three-factor model produced adequate-fit measures, whereas the one-factor model did not. The first factor, drive for size (DFS), consists of items concerning thoughts of being smaller and weaker than desired, or wishes to increase size and strength. This factor mirrors the MD criteria of preoccupation with muscularity [4]. The second factor, functional impairment (FI), contains items addressing the impairment associated with the main symptoms of MD (e.g., negative feelings associated with missing one or more workout days), and corresponds to the MD impairment criteria [4]. Finally, the third factor, appearance intolerance (AI), contains items related to negative beliefs about one’s body and resulting body exposure avoidance. This factor best matches the MD distress and avoidance criteria [4]. Our results replicate the three-factor structure described by original authors in a sample of 237 male weightlifters [16] and by [19] in 60 male competing bodybuilders. Furthermore, the results replicated the three-factor structure emerged in the Spanish [17,20,21] and German [22] versions, in cisgender lesbian women [15], and in Brazilian women [18]. Taken together, these findings support the notion that the MDDI subscales evaluate three distinct elements of the psychopathology and manifestations of MD [18]. Secondly, our results indicated that the factor structure of the MDDI was identical across groups, and that MDDI scores were scalar invariant across women practicing non-competing bodybuilding/powerlifting and women practicing physical exercise other than bodybuilding/powerlifting. 

Concerning internal consistency, adequate levels for both the MDDI total score and its scales have been found, in accordance with previous studies [16,17,21,22]. The results of the current study differed from results obtained by [19] with respect to the internal consistency of the AI scale, which emerged to be adequate in our study. Correlations between the MDDI total score and its scales emerged to be medium–large in both groups, in accordance with previous studies [16,19,22]. With respect to correlations among the MDDI scales, medium-small correlations emerged between the FI and the AI scales and between the FI and the DFS scales, whereas the DFS and the AI scales did not correlate in both groups, in accordance with the study by Zeeck and colleagues [22].

In addition, adequate evidence for convergent/divergent validity was found. With respect to the non-competing bodybuilding/powerlifting subgroup, significant positive and negative correlations (medium–large) emerged between the MDDI total score, the FI and the AI scales and measures of self-esteem (negative), social phobia symptoms (positive), and general distress (positive), in accordance with previous studies [16,19]. The DFS scale did not correlate with any of the other employed self-report measures in the bodybuilding/powerlifting subgroup. These results were somehow unexpected; however, it is worthy of note that previous studies reporting associations between the DFS subscale and self-esteem, social phobia symptoms, and general distress have been conducted only with males [16,19]. Indeed, the study by Galiana and colleagues [21] and by Zeeck and colleagues [22] did not assess associations among these constructs in females. Our results seem consistent with the notion that a drive for muscularity may not represent the core feature of MD symptoms in women: The cultural ideals for muscularity among men and women differed (i.e., lean and muscular shape with a well-developed chest and arms for men and thin, very toned, athletic body with flat, smooth muscles for women) [5,10,11,49,50,51]. 

With respect to the physical exercise subgroup, significant positive and negative correlations (medium–large) emerged between the MDDI total score, the FI and AI scales and measures of self-esteem (negative), social phobia symptoms (positive), and general distress (positive). Within this group, the DFS scale emerged to be positively associated with social phobia symptoms and general distress; however, correlations were very small (respectively, *r* = 0.13 and *r* = 0.17). 

Associations between MDDI scores and socio-demographic (age, education), BMI, and training features (starting date of training and weekly training) were also analyzed; results were similar in both groups. Taking into consideration only moderate and strong correlations (*rs* > 0.30), moderate positive associations between the AI scale and BMI emerged in both groups, whereas a negative pattern emerged between the DFS scale and BMI. Therefore, when BMI increases, women are characterized by negative thoughts about their body and, at the same time, when BMI decreases, women develop thoughts about being smaller and weaker than desired. The non-competing bodybuilding/powerlifting subgroup also showed positive moderate correlations between the FI scale and weekly training, in accordance with studies underlining the increased training when functional impairment due to MD symptomatology is high [1,2].

The non-competing bodybuilding/powerlifting subgroup, in accordance with studies conducted on males [1,7,8,52], showed higher levels of MDDI symptoms, higher desire to be more muscular (or beliefs of being smaller and weaker than desired), and more negative emotions when deviating from daily exercise and avoidance of social situations than women practicing physical exercise. These results are in accordance with studies underlining that MD symptoms differed in accordance with goals of physical exercise. Indeed, individuals involved in weight-training activities (such as individuals practicing resistance-training sports) may be at higher risk of developing MD symptoms than individuals involved in other type of physical exercise [1]. No differences emerged between groups on negative beliefs and anxiety associated with one’s body and physical appearance, in accordance with studies reporting high rates of body dissatisfaction and concerns about physical appearance in women [53,54]. 

Retest analysis supports the temporal stability of the MDDI after one month. So far, only two studies have investigated the stability of the MDDI across time; these studies found evidence of high temporal stability in male weightlifters [15,19]. In this context, our findings are relevant because this is the first study that has examined the temporal stability of the MDDI in physically active women, revealing its stability.

Despite these interesting results, the current study is characterized by several limitations. First of all, we relied on a relatively small sample of Italian women, which may limit the generalizability of our results. Related to this point, we did not ask for the ethnic background of participants. This represents a limitation of the study, since MD symptoms might differ among women with different ethnic backgrounds. Therefore, future studies should employ bigger samples including women with different ethnic backgrounds. Secondly, our recruitment strategy might have under-sampled individuals without internet capability and prevented the opportunity of conducting face-to-face interviews of participants. Third, women practicing bodybuilding and powerlifting were included in the same group (non-competing bodybuilding/powerlifting subgroup), despite these individuals are characterized by different sport goals (aesthetics *vs.* strength). Our decision was motivated by a previous study conducted with Italian male athletes [7] showing differences between bodybuilders and powerlifters only with respect to drive for size symptoms (no differences in other MD symptoms emerged).

## 5. Conclusions

These limitations notwithstanding, the present study provides evidence that MDDI scores are psychometrically valid in women practicing different types of physical activity. Establishing support for the validity and reliability of a measure in a sample that is distinct from those previously studied is a critical first step to using the measure in future research on the populations of interest [15] and it is crucial for the successful implementation of psychological interventions in different contexts. 

The current study makes an important contribution to the understanding of MD symptoms in women practicing different types of physical activity. In particular, our findings have important clinical implications; indeed, a new body ideal for women is emerging and taking the place of the thin ideal: a very toned, athletic body with flat, smooth muscles [10,11]. This new body ideal emphasizes strength [55] and, analogous to thin-ideal internalization, may negatively impact the psychological health of women [56]; therefore, the new emphasis on females’ body level of muscularity may represent a contribution to the growth of MD symptoms in women practicing physical activity, highlighting the importance of investigating this phenomenon also in women [9]. Since women practicing physical activity might be at risk of developing MD symptoms but the literature has focused almost entirely on men [9], these findings provide a hint for expanding MD studies to different context and populations, including the physical activity context. In accordance, our study is crucial for scholars and clinicians wishing to use the MDDI with Italian women practicing physical activity to identify women most at risk for MD. Such detection would better inform the development of psychological intervention that is specifically aimed at women with MD symptoms. In conclusion, our results showed that the MDDI represents a short and reliable measure, useful for the investigation of MD symptoms in physically active Italian women.

## Figures and Tables

**Table 1:** Main sample: Descriptive and comparisons between groups on demographics and training information.

	Overall Sample (*n* = 518) *M* (*DS*)/%	Non-Competing Bodybuilding/Powerlifting (*n* = 165) *M* (*DS*)/%	Physical Exercise (*n* = 353) *M* (*DS*)/%	*t*_(517)_/*χ*^2^_(4/6)_	*p*	*ηp* ^2^
Age	30.01 (8.05)	29 (7.78)	30.49 (8.16)	−1.96	0.05	-
Education	15.28 (2.87)	15.65 (3.08)	15.09 (2.74)	2.06	0.03	-
Body mass index	22.91 (3.95)	22.03 (3.05)	23.33 (4.26)	−3.87	*p* < 0.001	0.02
Marital Status						
Single	23.1	30.7	19.5			
In a relationship	32.4	36.7	30.3			
Married/cohabiting	42.2	31.9	47	17.32	0.002	-
Separated/divorced	2.1	0.6	2.8			
Widowed	0.2	0	0.3			
Occupation						
Student	28.8	28.3	29.2			
Full-time employed	29.9	26.5	31.4			
Part-time employed	11	14.5	9.3	6.67	0.35	-
Housewife	5.6	3.6	6.5			
Unemployed	8.9	8.4	9.1			
Other	15.8	18.6	14.4			
Starting date of exercise (in months)	34.22 (50.02)	29.69 (24.06)	35.93 (57.02)	−1.45	0.15	-
Weekly training (days)	4.18 (1.69)	3.89 (1.12)	4.30 (1.89)	−2.94	0.003	0.01

*Note.* significant *p* value ≤ 0.005.

**Table 2:** Retest sample: Descriptive.

	Retest Sample (*n* = 100) *M* (*DS*)/%	Non-Competing Bodybuilding/Powerlifting (*n* = 50) *M* (*DS*)/%	Physical Exercise (*n* = 50) *M* (*DS*)/%
Age	24.74 (4.53)	25.52 (5.69)	23.96 (2.80)
Education	15.18 (2.40)	14.52 (2.52)	15.84 (2.08)
Body mass index	21.15 (2.70)	21.81 (3.07)	20.48 (2.09)
Marital Status			
Single	31	36	26
In a relationship	54	38	70
Married/cohabiting	13	22	4
Separated/divorced	2	4	0
Widowed	0	0	0
Occupation			
Student	53	42	64
Full-time employed	21	30	12
Part-time employed	11	12	10
Housewife	1	2	0
Unemployed	4	6	2
Other	10	8	12
Starting date of exercise (in months)	48.71 (52.81)	38.18 (25.49)	59.24 (68.99)
Weekly training (days)	3.33 (1.11)	3.90 (0.76)	2.76 (1.12)

**Table 3:** Measurement of invariance across groups.

Model	*χ* ^2^	*df*	CFI	TLI	RMSEA	SRMR
Configural	353.206	124	0.956	0.945	0.085	0.093
Thresholds	376.606	137	0.954	0.948	0.083	0.093
Metric	388.126	145	0.954	0.951	0.080	0.093
Scalar	399.013	157	0.953	0.954	0.078	0.094

*Note.* CFI = comparative fit index; TLI = Tucker–Lewis index; RMSEA = root mean square error of approximation; SRMR = standardized root mean square residual.

**Table 4:** Correlations between MDDI, scores on other measures included in the study, age, education, BMI, starting date of exercise, and weekly training in the non-competing bodybuilding/powerlifting subsample (top diagonal) and in the physical exercise subsample (bottom diagonal).

	(1)	(2)	(3)	(4)	(5)	(6)	(7)	(8)	(9)	(10)	(11)	(12)
(1) MDDI_tot		0.81 **	0.59 **	0.57 **	−0.43 **	0.45 **	0.45 **	−0.17 *	−0.01	0.02	0.12	0.21 **
(2) MDDI_FI	0.80 **		0.31 **	0.28 **	−0.20 **	0.28 **	0.35 **	−0.07	0.04	−0.02	0.29 **	0.40 **
(3) MDDI_AI	0.73 **	0.38 **		−0.15	−0.60 **	0.52 **	0.42 **	−0.14	−0.04	0.46 **	0.03	0.01
(4) MDDI_DFS	0.43 **	0.19 **	−0.09		−0.05	0.09	0.11	−0.12	−0.03	−0.39 **	−0.08	0.01
(5) RSES	−0.49 **	−0.29 **	−0.54 **	−0.09		−0.63 **	−0.56 **	0.19 *	0.17 *	−0.14	−0.04	0.12
(6) SPS	0.45 **	0.26 **	0.46 **	0.13 **	−0.53 **		0.54 **	−0.24 **	−0.20 **	0.08	0.12	−0.01
(7) DASS-21	0.50 **	0.34 **	0.45 **	0.17 **	−0.68 **	0.52 **		−0.25 **	−0.03	0.12	−0.03	0.02
(8) Age	−0.21 **	−0.16 **	−0.07	−0.22 **	0.26 **	−0.27 **	−0.18 *		0.09	0.10	0.11	−0.02
(9) Education	−0.09	−0.03	−0.06	−0.09	0.07	−0.18 **	−0.08	0.09		0.09	0.12	0.06
(10) BMI	0.05	−0.04	0.41 **	−0.39 **	−0.13 *	0.07	0.04	0.22 **	0.02		−0.02	−0.06
(11) Months of Training	−0.07	0.04	−0.17 **	0.01	0.06	−0.14 *	−0.09	0.13 *	0.13*	−0.12		0.21 **
(12) Weekly Training (days)	0.06	0.19 **	−0.07	0.01	0.14 *	−0.12 *	−0.13 *	0.11 *	−0.12 *	−0.12 *	0.01	

*Note.* MDDI = muscle dysmorphic disorder inventory; FI = functional impairment; AI = appearance intolerance; DFS = drive for size; RSES = Rosenberg self-esteem scale; SPS = social phobia scale; DASS-21 = depression anxiety stress scale-21; BMI = body mass index. * *p* < 0.05; ** *p* < 0.01.

**Table 5:** Differences between groups on MDDI total score and scales.

	Non-Competing Bodybuilding/Powerlifting (*n* = 165) *M* (*DS*)	Physical Exercise (*n* = 353) *M* (*DS*)	*t*_(517)_	*p*	*ηp* ^2^
MDDI Total Score	31.52 (8.44)	27.44 (7.34)	5.34	*p* < 0.001	0.06
MDDI FI	11.32 (4.38)	8.59 (3.73)	6.92	*p* < 0.001	0.09
MDDI AI	11.09 (4.24)	11.89 (4.23)	−2.01	0.04	-
MDDI DFS	9.11 (4.21)	6.95 (2.88)	5.96	*p* < 0.001	0.08

*Note.* significant *p* value ≤ 0.005; MDDI = muscle dysmorphic disorder inventory; FI = functional impairment; AI = appearance intolerance; DFS = drive for size.

## Data Availability

The data presented in this study are available on reasonable request from the corresponding author (S.C.). The data are not publicly available because they report private information about participants.
